# Communication of Positive Lung Cancer Screening Findings and Receipt of Recommended Follow-up Care

**DOI:** 10.1001/jamanetworkopen.2023.20409

**Published:** 2023-06-22

**Authors:** Louise M. Henderson, Danielle D. Durham, Jason Long, Derek Lamb, Lindsay M. Lane, M. Patricia Rivera

**Affiliations:** 1 Department of Radiology, University of North Carolina, Chapel Hill; 2 Lineberger Comprehensive Cancer Center, University of North Carolina, Chapel Hill; 3 Department of Surgery, University of North Carolina, Chapel Hill; 4 Division of Pulmonary and Critical Care Medicine, University of Rochester Medical Center, Rochester, New York; 5 Wilmot Cancer Institute, University of Rochester, Rochester, New York

## Abstract

This cohort study evaluates associations of communication methods and content of positive lung cancer screening findings with receipt of recommended follow-up care.

## Introduction

Lung cancer screening (LCS) with low-dose computed tomography (LDCT) reduces lung cancer mortality among individuals with high risk, yet 13% to 25% of screened patients have a positive result.^1,2^ The Lung CT Screening Reporting and Data System (Lung-RADS) was developed to standardize reporting of LCS results and recommended follow-up according to the level of suspicion of the findings,^3^ yet limited evidence exists regarding how positive LCS results are communicated to patients.^4^ We evaluated the association of communication methods and content of positive LCS findings with receipt of recommended follow-up care.

## Methods

This cohort study was approved by the University of North Carolina institutional review board under a waiver of the Health Insurance Portability and Accountability Act and informed consent because the study demonstrated no more than minimal risk to participants or their privacy. This study followed the Strengthening the Reporting of Observational Studies in Epidemiology (STROBE) reporting guideline.

Details of this cohort have been previously published.^5^ We included patients who underwent LCS at 5 North Carolina sites from January 2015 to February 2020 and had a positive screen result, defined as Lung-RADS 3 (probably benign, recommended follow-up LDCT within 6 months), Lung-RADS 4A (suspicious, recommended follow-up LDCT or positron emission tomography and CT [PET/CT] within 3 months), or Lung-RADS 4B or 4X (very suspicious, recommended follow-up chest CT with or without contrast, PET/CT, or tissue sampling to occur immediately). We extended our prior work by abstracting electronic health record (EHR) data on the documented communication method and content of results to patients. Communication methods were classified into 4 mutually exclusive groups: (1) received mailed letter, voicemail message, text message, email, or online portal message; (2) telephone conversation with clinician; (3) in-person office visit (with any other method); and (4) no communication documented. If multiple communication methods were used, we classified the most intense method (3 > 2 > 1 > 4). Communication content included: (1) abnormal or suspicious findings; (2) benign findings; (3) specific follow-up needed (LDCT, CT, PET/CT, biopsy); and (4) specific timing for follow-up. We determined if the patient underwent recommended follow-up per Lung-RADS and used a 1-month follow-up for Lung-RADS 4B or 4X.^5^ We compared the communication method and content by Lung-RADS using χ^2^ tests. We determined if the communication method or content was associated with receipt of recommended follow-up care, stratified by Lung-RADS and adjusted for patient age, sex, race, smoking status, and site using multivariable logistic regression excluding 15 patients with no documented communication. All analyses were conducted from September 2022 to March 2023 using SAS statistical software version 9.4 (SAS Institute). *P* values were 2-sided, and statistical significance was set at *P* = .05.

## Results

Among 685 included patients (363 patients [53.0%] with Lung-RADS 3; 194 patients [28.3%] with Lung-RADS 4A; 128 patients [18.7%] with Lung-RADS 4B or 4X), the communication method and content of positive LCS results varied by Lung-RADS (Table 1). Results were more commonly communicated by letter, voicemail, text, email, or online portal messages to patients with Lung-RADS 3 findings vs those with Lung-RADS 4A findings, or Lung-RADS 4B or 4X findings (Table 1). Lung-RADS 3 communications were more likely to mention benign findings (65.8%), while communications were more likely to mention abnormal or suspicious findings for Lung-RADS 4A (49.0%) or Lung-RADS 4B or 4X (72.7%) (*P* < .001). Specific follow-up timing was less frequently mentioned in Lung-RADS 4B or 4X communications (46.9%) than in Lung-RADS 3 (95.9%) or Lung-RADS 4A (82.0%) communications (*P* < .001) (Table 1).

**Table 1.** Method and Content of Communication of Positive Lung Cancer Screening Findings by Lung-RADS Assessment Category, 2015-2020

Communication	Patients, No. (%)			*P* value
	Lung-RADS 3 (n = 363)	Lung-RADS 4A (n = 194)	Lung-RADS 4B or 4X (n = 128)	
Method				
Letter, voicemail, text, email, or portal	96 (26.4)	39 (20.1)	22 (17.2)	.04
Phone call conversation	112 (30.9)	80 (41.2)	57 (44.5)	
Office visit	140 (38.6)	68 (35.1)	47 (36.7)	
None documented	15 (4.1)	7 (3.6)	2 (1.6)	
Content^a^				
Mention benign finding				
Yes	239 (65.8)	11 (5.7)	3 (2.3)	<.001
No	124 (34.2)	183 (94.3)	125 (97.7)	
Mention abnormal or suspicious finding				
Yes	62 (17.1)	95 (49.0)	93 (72.7)	<.001
No	304 (83.7)	99 (51.0)	35 (27.3)	
Mention specific follow-up test needed				
Yes	346 (95.3)	179 (92.3)	119 (93.0)	.30
No	17 (4.7)	18 (9.3)	9 (7.0)	
Mention specific follow-up timing needed				
Yes	348 (95.9)	159 (82.0)	60 (46.9)	<.001
No	15 (4.1)	35 (18.0)	68 (53.1)	
Screened patient stated seeking a second opinion				
Yes	22 (6.1)	33 (17.0)	29 (22.7)	<.001
No	341 (93.9)	161 (83.0)	99 (77.3)	

Abbreviation: Lung-RADS, Lung CT Screening Reporting & Data System.

^a^ Individuals could have more than 1 communication content documented (eg, mention of abnormal finding and follow-up timing).

Patients with Lung-RADS 3 findings whose communication mentioned benign findings were less likely to receive recommended follow-up vs those with no mention of benign findings (adjusted odds ratio [aOR], 0.54; 95% CI, 0.32-0.90) (Table 2). Among patients with Lung-RADS 4A findings, those who spoke with a clinician by phone were 3 times more likely to receive recommended follow-up compared with those whose results were communicated by letter, voicemail, text, email, or online portal (aOR, 3.15; 95% CI, 1.30-7.60) (Table 2).

**Table 2.** Association of Method and Content of Communication on Receipt of Guideline-Recommended Follow-up Care, by Lung-RADS Assessment, 2015-2020

Communication variable	Lung-RADS 3: follow-up within 6 mo			Lung-RADS 4A: follow-up within 3 mo			Lung-RADS 4B or 4X: follow-up within 1 mo		
	No. (%)		aOR (95%CI)^a^	No. (%)		aOR (95%CI)^a^	No. (%)		aOR (95%CI)^a^
	Yes	No		Yes	No		Yes	No	
Total	105 (30.2)	243 (69.8)	NA	91 (48.7)	96 (51.3)	NA	85 (67.5)	41 (32.5)	NA
Method of communication									
Letter, voicemail, text, email, or portal	27 (25.7)	69 (28.4)	1 [Reference]	14 (15.4)	25 (26.0)	1 [Reference]	11 (12.9)	11 (26.8)	1 [Reference]
Phone call conversation	36 (34.3)	76 (31.3)	1.12 (0.58-2.15)	48 (52.7)	32 (33.3)	3.15 (1.30-7.60)	42 (49.4)	15 (36.6)	1.65 (0.52-5.29)
Office visit	42 (40.0)	98 (40.3)	1.02 (0.54-1.93)	29 (31.9)	39 (40.6)	1.60 (0.66-3.91)	32 (37.6)	15 (36.5)	1.55 (0.47-5.14)
Communication content includes *benign* finding									
Yes	60 (57.1)	178 (73.3)	0.53 (0.32-0.90)	4 (4.4)	7 (7.3)	NA	0	3 (7.3)	NA
No	45 (42.9)	65 (26.8)	1 [Reference]	87 (95.6)	89 (92.7)	NA	85 (100)	38 (92.7)	NA
Communication content includes *abnormal* or *suspicious* finding									
Yes	25 (23.8)	37 (15.2)	1.51 (0.81-2.80)	51 (56.0)	41 (42.7)	1.36 (0.70-2.62)	66 (77.7)	26 (63.4)	1.46 (0.57-3.75)
No	80 (76.2)	206 (84.8)	1 [Reference]	40 (44.0)	55 (57.3)	1 [Reference]	19 (22.4)	15 (36.6)	1 [Reference]
Communication content includes specific follow-up timing needed									
Yes	102 (97.1)	235 (96.7)	NA	70 (76.9)	84 (87.5)	0.56 (0.23-1.38)	38 (44.7)	22 (53.7)	0.92 (0.39-2.20)
No	3 (2.9)	8 (3.3)	NA	21 (23.1)	12 (12.5)	1 [Reference]	47 (55.3)	19 (46.3)	1 [Reference]

Abbreviations: aOR, adjusted odds ratio; Lung-RADS, Lung CT Screening Reporting & Data System; NA, not included in model.

^a^ Adjusted for age, race, sex, smoking status (current, former), and imaging site.

## Discussion

This cohort study found that as the lung cancer suspicion level increased, the communication method shifted from passive to more active modes, consistent with survey data showing clinicians and patients prefer verbal communication modes to discuss suspicious findings.^6^ The Lung-RADS lexicon uses *probably benign* terminology for Lung-RADS 3. This language was included in 66% of communications among Lung-RADS 3 patients and patients with communications that mentioned *benign* were significantly less likely to undergo recommended follow-up, suggesting other terms should be considered for communicating Lung-RADS 3 results. Patients with Lung-RADS 4A findings who talked with a clinician by phone were 3 times more likely to receive recommended follow-up than patients with passive follow-up, highlighting an area of improved care. For Lung-RADS 4B or 4X findings, the communication content was less likely to include the specific follow-up timing interval, likely due to the Lung-RADS lexicon using the term *immediate*. Our study is limited to a population from a single geographic region, which may not be generalizable to the entire US, and the use of EHR data, which may be incomplete. Our study identifies communication areas to target to increase appropriate follow-up care among patients with positive LCS results.

## Notes

Supplement.
Data Sharing Statement
