# Supplementary material for: Communication of Positive Lung Cancer Screening Findings and Receipt of Recommended Follow-up Care
Source: JAMA Netw Open. 2023 Jun 22;6(6):e2320409. doi: 10.1001/jamanetworkopen.2023.20409 (PMC10288328; doi:10.1001/jamanetworkopen.2023.20409)
Supplement: Supplement. — Data Sharing Statement [file jamanetwopen-e2320409-s001.pdf]

Data Sharing Statement

Henderson. Communication of Positive Lung Cancer Screening Findings and Receipt of Recommended Follow-up Care. *JAMA Netw Open*. Published June 22, 2023. doi:10.1001/jamanetworkopen.2023.20409

Data
Data available: No

Additional Information
Explanation for why data not available: The data used in this study has restrictions on sharing per the data use agreements from the original owners of the data. Access to the data may be approved once a proposal for a specific study question has been reviewed by the original data owners.
